# Model-based comparison of subcutaneous versus sublingual apomorphine administration in the treatment of motor fluctuations in Parkinson’s disease

**DOI:** 10.1007/s10928-024-09914-x

**Published:** 2024-04-05

**Authors:** Azmi Nasser, Roberto Gomeni, Gianpiera Ceresoli-Borroni, Lanyi Xie, Gregory D. Busse, Zare Melyan, Jonathan Rubin

**Affiliations:** 1 Formerly with Supernus Pharmaceuticals, Inc., 9715 Key West Ave, Rockville, MD 20850 USA; 2 PharmacoMetrica, Lieu-dit Longcol, La Fouillade, France; 3 Supernus Pharmaceuticals, Inc., Rockville, MD USA

**Keywords:** Parkinson’s disease, Motor fluctuations, Off-episodes, Apomorphine, Pharmacokinetics

## Abstract

The objective of this study was to compare the effectiveness of subcutaneous (SC) and sublingual (SL) formulations of apomorphine for the treatment of motor fluctuations in Parkinson’s disease using a pharmacokinetics (PK)/pharmacodynamics (PD) modeling approach. The PK of SC and SL apomorphine are best described by a one-compartment model with first-order absorption and a two-compartment model with delayed absorption, respectively. The PK/PD model relating apomorphine plasma concentrations to the Unified Parkinson’s Disease Rating Scale (UPDRS) motor scores was described by a sigmoidal E_max_ model assuming effective concentration = drug concentration in an effect compartment. Apomorphine concentrations and UPDRS motor scores were simulated from the PK/PD models using 500 hypothetical subjects. UPDRS motor score change from baseline was evaluated using time to clinically relevant response, response duration, area under the curve, maximal response, and time to maximal response. Higher doses of each apomorphine formulation were associated with shorter time to response, longer response duration, and greater maximal response. Although the mean maximal responses to SC and SL apomorphine were comparable, the time to response was four times shorter (7 vs. 31 min) and time to maximal response was two times shorter (27 vs. 61 min) for 4 mg SC vs. 50 mg SL. Thus, faster onset of action was observed for the SC formulation compared to SL. These data may be useful for physicians when selecting “on demand” therapy for patients with Parkinson’s disease experiencing motor fluctuations.

**Supplementary Information:**

The online version contains supplementary material available at 10.1007/s10928-024-09914-x.

## Introduction 

Oral levodopa remains the mainstay of symptomatic therapy for Parkinson’s disease, although its prolonged use is associated with the development of motor complications such as motor fluctuations and dyskinesia [1]. Approximately 40% of patients develop motor fluctuations and dyskinesia within 4–6 years and 70% after ≥ 9 years of treatment with levodopa [2]. Patients can experience multiple OFF-episodes per day with cumulative daily OFF-time accounting for up to 50% of a patient’s waking day [3, 4], with significant impact on quality of life [5, 6]. There are three main approaches to managing levodopa-related motor complications; the addition of oral adjuncts, continuous drug delivery (e.g. infusion), and the use of ‘on demand’ medications designed to rapidly abort OFF episodes. Apomorphine, a short-acting dopamine (D)_1_ and D_2_ receptor agonist, is the only medication proven to have an efficacy equal to that of levodopa in reducing motor symptoms [7]; it has a more rapid onset of action than levodopa, but a shorter effect duration [8]. Two types of ‘on demand’ apomorphine therapy have been approved for use in the US, namely the subcutaneous (SC) formulation and the sublingual (SL) formulation [9, 10]; the SL formulation is no longer commercially supported in the US. The recommended apomorphine dose for SC administration is 2 mg to 6 mg and 10 mg to 30 mg for SL administration [9, 10].

Both ‘on demand’ apomorphine formulations were developed to overcome the limitations of oral apomorphine, which has a short half-life and undergoes extensive metabolism in the gastrointestinal tract, and both have demonstrated efficacy in clinical trials [8]. However, factors such as formulation effectiveness and route of administration-related adverse effects, may influence patient experience and clinical utility. In terms of efficacy, time to onset, reliability of effect, and duration of effect sufficient to last until onset of regularly scheduled medication are important features for an on-demand therapy [11, 12]. From a safety perspective, SC administration of apomorphine may cause injection site reactions such as bruising (16%), granulomas (4%) or itching (2%), but these typically do not lead to discontinuation (≤ 5% of patients) [9]. In contrast, SL apomorphine may cause oropharyngeal irritation, including tissue swelling (~ 15%), pain (~ 13%), ulceration (~ 7%) or erythema (~ 7%), that is a more frequent cause of discontinuation (~ 17% of patients in a 12-week trial) and can prevent continued use of the product [10, 13].

The bioavailability of the two on demand formulations are very different. The SC formulation is 100% bioavailable with similar absorption, volume of distribution, plasma clearance, and half-life characteristics to intravenous infusion [14]. However, several factors can influence the SC absorption, such as state of the skin at the injection site, as well as injection depth and volume [8]. The SL apomorphine, on the other hand, has bioavailability of only 17%–18%, which can be explained by nonexclusive absorption via the SL or buccal routes, when part of it is being swallowed and absorbed in the stomach [15, 16]. These differences in PK characteristics can lead to differences in the time course of the effect intensity between different formulations [17].

The models describing PK characteristics of SC and SL apomorphine formulations, as well as the PK/PD model of apomorphine, have been previously developed [15, 18, 19]. The objective of this analysis was to compare the effectiveness of the SC and SL formulations of apomorphine for the treatment of motor fluctuations in Parkinson’s disease using a PK/PD modeling approach.

## Methods

To evaluate clinical response associated with the SC and SL administration of apomorphine, we used the PK/PD model relating the circulating apomorphine concentrations to the changes in the Unified Parkinson’s Disease Rating Scale (UPDRS) motor scores (UPDRS Part III), a standardly used rating scale in Parkinson’s disease clinical trials [20]. Part III of the scale is a clinical evaluation of motor symptoms with score changes from full OFF to full ON exceeding 10 or more points [21, 22]. The PK model developed by the FDA for SC apomorphine, the recently published PK model for SL apomorphine, and the FDA-developed PK/PD model of apomorphine were used in the analysis [15, 18, 19].

The recommended dose for the SC administration of apomorphine ranges from 2 to 6 mg, and the recommended dose for the SL administration ranges from 10 to 30 mg [9, 10]. Given the differences in the PK characteristics between two formulations [15, 18, 19], the PK/PD relationships were explored at a range of dose levels: 1 mg, 2 mg, 3 mg, and 4 mg for the SC administration and 20 mg, 30 mg, 40 mg, and 50 mg for the SL administration. The goal in including higher than recommended doses for SL and lower than recommended doses for SC was to demonstrate that the results of the model apply even at the highest levels of exposure for SL and lowest levels of exposure for SC. This comparison serves to advantage the SL dosage form and disadvantage the SC dosage form by comparing efficacy for what could be considered supratherapeutic doses of SL and subtherapeutic doses of SC. The expected apomorphine plasma concentrations and UPDRS motor scores (Part III) for the SC and the SL formulations of apomorphine were simulated for 500 subjects for each dose level.

To evaluate the impact of the inter-individual variability (IIV) in the predicted PK and in the PK/PD time course, three levels of IIV were considered (with 15%, 30%, and 45% coefficient of variation [CV]). Simulations were performed for each formulation, dose level, and level of IIV.

The clinical benefit was evaluated by comparing parameters qualifying the response to treatment (change from baseline UPDRS motor score) estimated for SC and SL administration over the interval 0 to 90 min post-dose using trial simulations. These parameters included: 1) time to clinically relevant response (defined as a change from baseline in the UPDRS motor score of − 3.25 units [23]); 2) response duration (a time period during which the change from baseline in the UPDRS motor score remained ≥  − 3.25 units); 3) area under the curve for the change from baseline in the UPDRS motor score; 4) maximal change from baseline in the UPDRS motor score; and 5) time to the maximal change from baseline of the UPDRS motor score.

The simulations were conducted using NONMEM® software (version 7.4, ICON Development Solutions). The analyses were conducted using R (version 4.0.0); the summary statistics were generated using SAS® (version 9.4).

### PK model for SC apomorphine

**Fig. 1:**
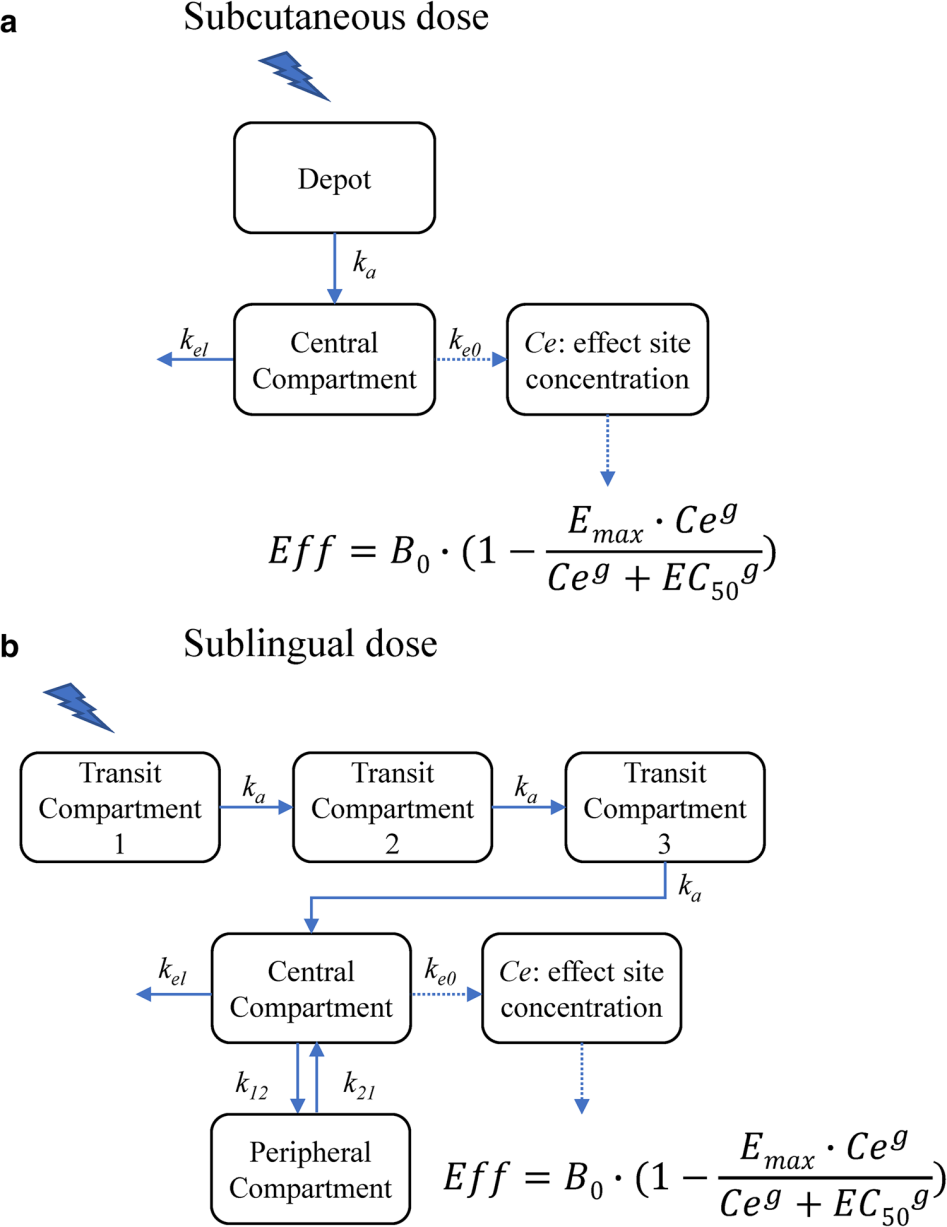
**a** Schematic of the apomorphine PK model following subcutaneous administration. Abbreviations:* B*_*0*_, baseline motor score; *Ce*, drug concentration at the site of action; *EC*_*50*_, drug concentration that causes 50% of the maximum effect; *E*_*max*_, maximum inhibitory effect; *g*, Hill’s sigmoid coefficient; *k*_*a*_, first-order absorption constant;* k*_*e0*_, first-order plasma-effect-site equilibration rate constant; *k*_*el*_, elimination constant; PK, pharmacokinetics. **b** Schematic of the apomorphine PK model following sublingual administration. Abbreviations:* B*_*0*_, baseline motor score; *Ce*, drug concentration at the site of action; *EC*_*50*_, drug concentration that causes 50% of the maximum effect; *E*_*max*_, maximum inhibitory effect; *g*, Hill’s sigmoid coefficient; *k*_*12*_ and *k*_*21*_, first-order transfer rate constants between the central and peripheral compartments; *k*_*a*_, first-order absorption constant;* k*_*e0*_, first-order plasma-effect-site equilibration rate constant; *k*_*el*_, elimination constant; PK, pharmacokinetics

The PK time course of apomorphine after SC administration was best described by a one-compartment model with first-order absorption and elimination processes (Fig. 1a) [18, 19]. The model was defined by two differential equations:

$$\begin{array}{l}\frac{dA}{dt}=-k_a\cdot A\\\\\frac{dC}{dt}=k_a\cdot A-k_{el}\cdot C\\\\Cp=\frac C{V/F}\end{array}$$

*A* and *C* are the amounts of drug in the depot and central compartments, *k*_*a*_ and *k*_*el*_ are the first-order absorption and elimination rate constants, *V/F* is the volume of distribution, *F* is the bioavailability (assumed equal to one), and *Cp* is the drug concentration in the central compartment.

### PK model for SL apomorphine

The PK time course of apomorphine after a SL administration was best described by a two-compartment model with a delayed absorption process, which was described by a transit compartments model, a first-order distribution from/to a peripheral compartment, and a first-order elimination rate constant (Fig. 1b) [15]. The model was defined by five differential equations:

$$\begin{array}{l}\frac{{dT}_1}{dt}=-k_a\cdot T_1\\\\\frac{{dT}_2}{dt}=k_a\cdot\left(T_1-T_2\right)\\\\\begin{array}{l}\frac{{dT}_3}{dt}=k_a\cdot\left(T_2-T_3\right)\\\\\frac{dC}{dt}=k_a\cdot T_3-k_{el}\cdot C-k_{12}\cdot C+k_{21}\cdot P\\\\\begin{array}{l}\frac{dP}{dt}=k_{12}\cdot C-k_{21}\cdot P\\\\Cp=\frac C{V/F}\end{array}\end{array}\end{array}$$

*T*_*1*_, *T*_*2*_, and *T*_*3*_ are the amount of drug in the transit compartments, *C* and *P* are the amount of drug in the central and peripheral compartments, *k*_*a*_ and *k*_*el*_ are the first-order absorption and elimination rate constants, *k*_*12*_ and *k*_*21*_ are the first-order transfer rate constants between the central and peripheral compartments, *V/F* is the volume of distribution, *F* is the bioavailability, and *Cp* is the drug concentration in the central compartment.

### PK/PD model

The model relating the apomorphine plasma concentration to the UPDRS motor scores was developed based on the observation that the UPDRS time course was not directly related to the time course of apomorphine plasma concentrations. Therefore, a "link model" approach was used to implement the PK/PD model and to estimate the drug concentration in the effect-site compartment [24]. The basic assumption of this model is that the rate of drug distribution to/from the hypothetical effect site determines the rate of onset/offset of the effect.

$$\begin{array}{c}\frac{dCe}{dt}={k}_{e0}\cdot \left(Cp-Ce\right)\\ Eff={B}_{0}\cdot \left(1-\frac{{E}_{max}\cdot {Ce}^{g}}{{Ce}^{g}+{{EC}_{50}}^{g}}\right)\end{array}$$

*B*_*0*_ is the baseline motor score, *E*_*max*_ is the maximum inhibitory effect, *Cp* is the apomorphine plasma concentration predicted by the PK model, *Ce* is the drug concentration at the site of action, *EC*_*50*_ is the drug concentration that causes 50% of the maximum effect, *g* is the Hill’s sigmoid coefficient, and *k*_*e0*_ is the first-order plasma-effect-site equilibration rate constant.

The mean population PK parameters used for these simulations were estimated in the reference publications describing the respective PK model for each formulation (Table 1) [15, 18, 19]. There were no covariates used in the simulations.

**Table 1:** Mean population PK values estimated in the reference publications for the SL and SC formulations of apomorphine [15, 18, 19]

PK parameter	Subcutaneous	Sublingual
CL/F (L/h)	191	80.7
V/F (L)	153	438
K_a_ (h^−1^)	14.9	6.58
K_12_ (h^−1^)	-	0.613
K_21_ (h^−1^)	-	0.0048
F (%)	1	0.206

Abbreviations: CL/F, drug clearance; F, bioavailability; k_12_, elimination rate constant of drug from central compartment to peripheral compartment; k_21_, elimination rate constant of drug from peripheral compartment to central compartment; k_a_, absorption rate constant; PK, pharmacokinetics; V/F, volume of distribution

Additionally, the mean population PK/PD parameters describing the longitudinal change in the UPDRS motor score as a function of apomorphine exposure estimated in the reference publications are summarized in Table 2 [18, 19].

**Table 2:** Mean population PK/PD values describing the longitudinal change in the UPDRS motor score as a function of the apomorphine exposure estimated in the reference publications [18, 19]

PK/PD parameter	Estimate
k_e0_ (h^−1^)	5.36
EC_50_ (ng/mL)	10.7
B_0_	24.3
E_max_	1
g	3

Abbreviations:* B*_*0*_, baseline motor score; *EC*_*50*_, drug concentration that causes 50% of the maximum effect; *E*_*max*_, maximum inhibitory effect; *g*, Hill’s sigmoid coefficient; *k*_*e0*_, first-order plasma-effect-site equilibration rate constant; PD, pharmacodynamics; PK, pharmacokinetics. UPDRS, Unified Parkinson’s Disease Rating Scale

## Results

### PK simulations

**Fig. 2:**
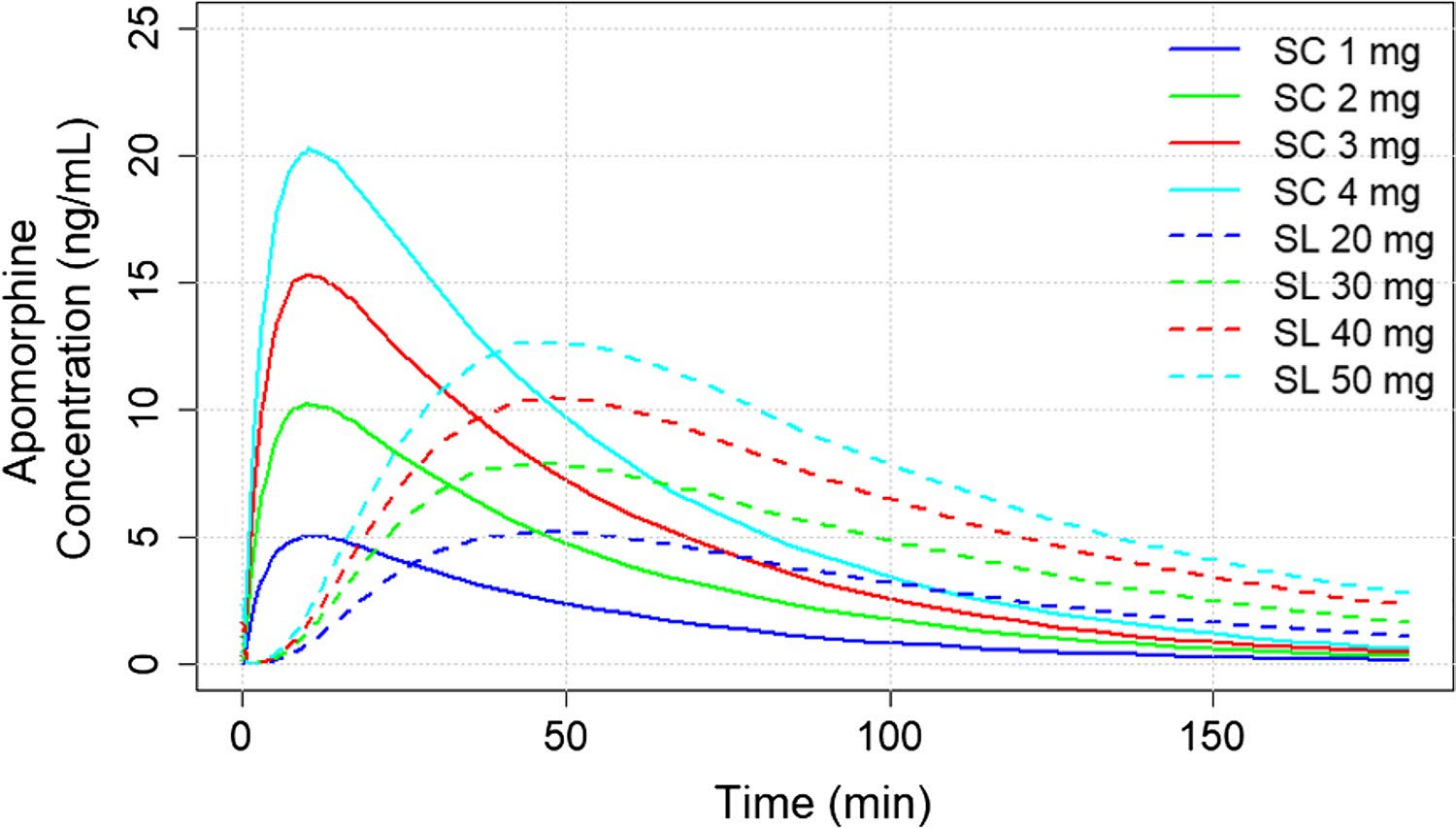
Mean apomorphine concentration–time profiles for the 1 mg, 2 mg, 3 mg, and 4 mg SC administration and 20 mg, 30 mg, 40 mg, and 50 mg SL administration. The recommended dose for SC administration is 2 mg to 6 mg and 10 mg to 30 mg for SL administration [9, 10]. Abbreviations: min, minute; SC, subcutaneous; SL, sublingual

The mean apomorphine plasma concentration–time profiles for the SC and SL administration simulations are displayed in Fig. 2, demonstrating a dose-dependent increase in plasma concentration for both formulations.

The mean area under the concentration–time curve (AUC_0-90_), maximum plasma concentration (C_max_), and time to maximum plasma concentration (T_max_) values by dose at 30% CV are shown in Table 3. Greater AUC_0-90_ and C_max_ values, and shorter T_max_ were found for 2 to 4 mg doses of SC apomorphine versus 30 to 50 mg doses of SL apomorphine.

**Table 3:** Mean apomorphine AUC_0-90_, C_max_, and T_max_ values for SC and SL formulations by dose, at 30% inter-individual variability

	Subcutaneous		Sublingual	
	Dose, mg	PK parameter, mean ± SD	Dose, mg	PK parameter, mean ± SD
AUC_0-90,_ ng*h/mL	1*	224.49 ± 58.90	20	58.62 ± 33.91
	2	451.08 ± 121.06	30	91.19 ± 50.70
	3	682.48 ± 177.69	40*	119.02 ± 68.44
	4	898.98 ± 236.12	50*	147.37 ± 86.53
C_max_	1*	5.28 ± 1.38	20	5.59 ± 1.65
	2	10.63 ± 2.81	30	8.56 ± 2.63
	3	16.05 ± 4.18	40*	11.37 ± 3.39
	4	21.13 ± 5.46	50*	13.96 ± 4.51
T_max_	1*	11.10 ± 2.74	20	47.57 ± 11.39
	2	11.13 ± 2.91	30	45.88 ± 12.39
	3	10.93 ± 2.68	40*	47.78 ± 11.72
	4	11.09 ± 2.71	50*	47.16 ± 12.06

*Falls outside the recommended dosing. The recommended dose for SC administration is 2 mg 6 mg and 10 mg to 30 mg for SL administration [9, 10].

Abbreviations: AUC_0-90_, area under the concentration–time curve for 0 to 90 min; C_max_, maximum plasma concentration; PK, pharmacokinetics; SD, standard deviation; T_max_, time to maximum plasma concentration

### PK/PD simulations

**Fig. 3:**
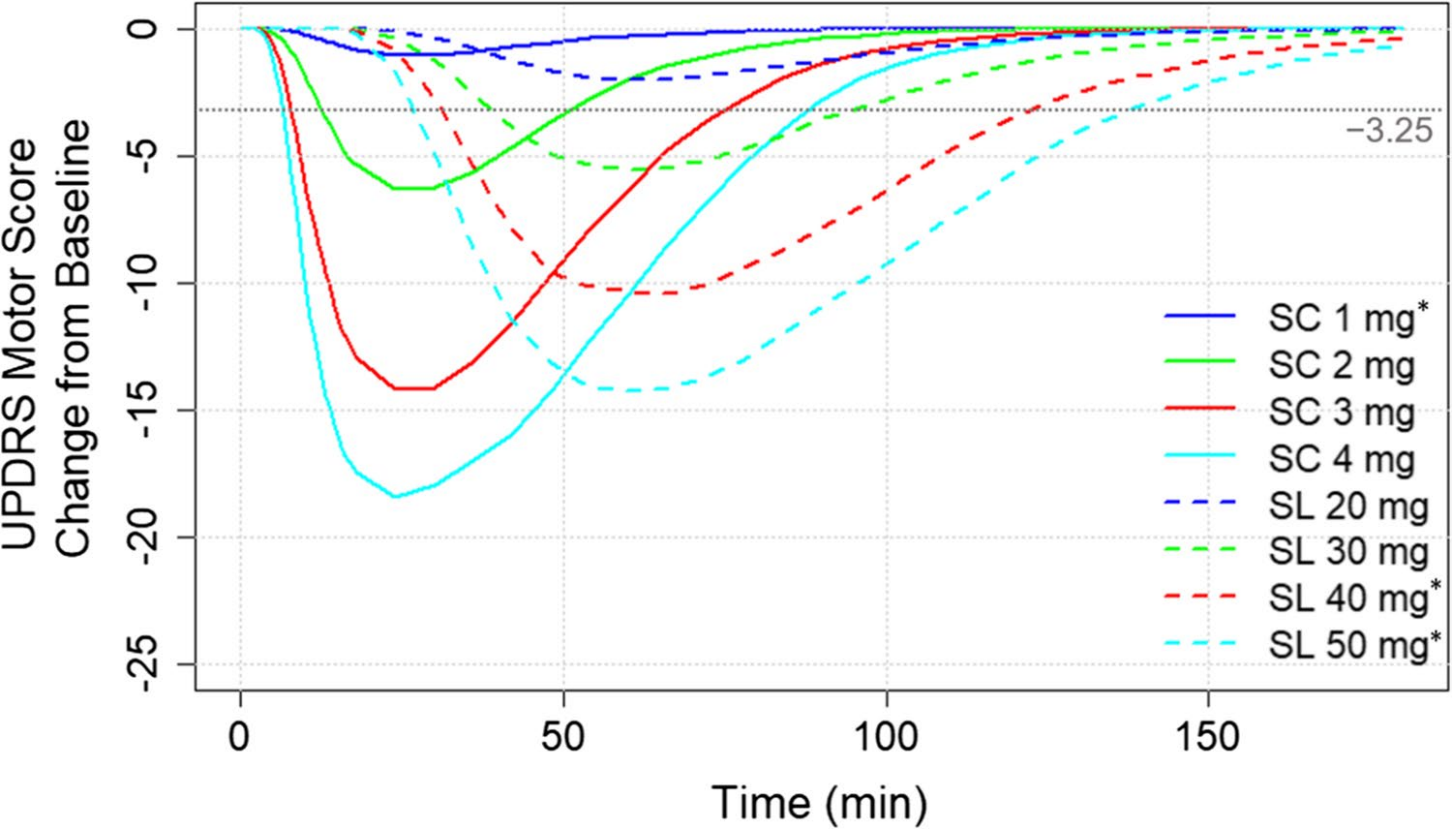
Mean longitudinal change from baseline profiles in the UPDRS motor scores for the 1 mg, 2 mg, 3 mg, and 4 mg SC administration and 20 mg, 30 mg, 40 mg*, and 50 mg* SL administration. The horizontal dashed line represents the MCIC for the UPDRS. The MCIC value [23] was used to define the time to response as the time to achieve MCIC and the duration of response as the time elapsed below the MCIC line. *Falls outside the recommended dosing. The recommended dose for SC administration is 2 mg to 6 mg and 10 mg to 30 mg for SL administration [9, 10]. Abbreviations: MCIC, minimum clinically important change; min, minute; SC, subcutaneous; SL, sublingual; UPDRS, Unified Parkinson’s Disease Rating Scale

The model-predicted mean longitudinal change from baseline profiles in the UPDRS motor scores for SC and SL administration at the doses of 1 mg, 2 mg, 3 mg, and 4 mg, and 20 mg, 30 mg, 40 mg, and 50 mg, respectively, are plotted in Fig. 3, demonstrating dose-dependent changes in the UPDRS motor scores for both formulations.

The mean values qualifying the response to the treatment with the SC and SL apomorphine by dose at 30% CV are shown in Table 4 (for the full list of descriptive statistics at 15%, 30%, and 45% CV, see Supplemental Table 1). Higher doses of each apomorphine formulation were associated with shorter time to response, longer response duration, and greater maximal response. The time to maximal response values were similar across different doses for each formulation. Shorter mean time to response, greater mean area under the effect curve (AUEC), and shorter mean time to maximal response were observed for the SC formulation compared to SL, while the mean maximum response values were comparable. For example, mean time to response was more than four times shorter for 4 mg SC apomorphine compared with 50 mg SL apomorphine (7 min vs. 31 min), and mean time to maximal response was more than two times shorter (27 min vs. 61 min).

**Table 4:** Mean parameters qualifying response to treatment for SC and SL formulations by dose, at 30% inter-individual variability

	Subcutaneous		Sublingual	
	Dose, mg	Parameter, mean ± SD	Dose, mg	Parameter, mean ± SD
Time to response, min	1	14.93 ± 5.06	20	39.88 ± 11.82
	2	12.27 ± 5.33	30	36.82 ± 11.11
	3	8.65 ± 3.60	40	33.40 ± 10.78
	4	7.34 ± 3.34	50	30.53 ± 9.63
Duration of response, min	1	28.82 ± 16.49	20	43.76 ± 15.42
	2	44.49 ± 19.18	30	48.98 ± 14.85
	3	64.59 ± 17.05	40	55.40 ± 12.40
	4	72.23 ± 14.69	50	58.63 ± 10.78
AUEC_0-90_	1	109.16 ± 88.64	20	11.77 ± 11.86
	2	176.98 ± 141.75	30	16.22 ± 17.18
	3	321.12 ± 188.65	40	23.98 ± 23.23
	4	428.95 ± 211.84	50	31.37 ± 29.40
Maximal response	1	− 6.05 ± 3.13	20	− 7.07 ± 3.47
	2	− 9.12 ± 4.64	30	− 9.28 ± 4.85
	3	− 14.46 ± 4.97	40	− 12.50 ± 5.31
	4	− 17.48 ± 4.71	50	− 14.83 ± 5.46
Time to maximal response, min	1	25.06 ± 5.35	20	59.93 ± 12.33
	2	26.49 ± 5.68	30	60.02 ± 11.36
	3	26.91 ± 5.65	40	61.77 ± 11.62
	4	27.09 ± 5.75	50	61.39 ± 11.70

The time to response is the time to achieve MCIC (Fig. 3), and the duration of response is the time elapsed below the MCIC (i.e., with a reduction of at least 3.5 points).

Abbreviations: AUEC_0-90_, area under the effect curve for 0 to 90 min; MCIC, minimum clinically important change; min, minute; SD, standard deviation

## Discussion

Comparison of two apomorphine formulations revealed significant differences in the time course of the change from baseline in the UPDRS motor scores, demonstrating shorter time to response and shorter time to maximal response for SC administration compared to SL. The differences between these two routes of administration are likely to be related to the differences in the absorption routes of SC and SL apomorphine and are apparent even when comparing lower than recommended doses of SC apomorphine with higher than recommended doses for SL apomorphine.

Following SC administration into the fatty area of the abdomen, the subcutis (the layer of skin directly below the dermis and epidermis, collectively referred to as the cutis), apomorphine is rapidly absorbed and the T_max_ ranges from 10 to 40 min. Apomorphine exhibits linear PK over a dose range between 2 and 8 mg following a single SC injection in patients with idiopathic Parkinson’s disease. The bioavailability of SC apomorphine is equal to that of an intravenous administration [9, 18, 19]. This is different from the oral administration route, which has a low bioavailability due to a first-pass metabolism of the drug [25].

Apomorphine SL film consists of two layers: (1) a drug layer designed for stability, rapid diffusion, and maximal bioavailability, and (2) a buffer layer designed to neutralize acid generation and enhance drug permeability [26]. Following SL administration, the T_max_ of apomorphine ranges from 30 to 60 min, with a bioavailability of ~ 20% [10, 15]. After SL administration, part of the drug will be swallowed and absorbed through the gastrointestinal wall, which explains the low bioavailability compared to complete drug absorption after SC administration. Apomorphine exhibits less than a dose proportional increase in exposures over a dose range of 10 mg to 35 mg following a single SL administration in patients with Parkinson's disease [10, 15].

The lack of dose proportionality observed in SL apomorphine exposure appears to be more pronounced in patients with Parkinson’s disease compared to healthy subjects, which may be attributed to extrinsic factors, such as SL film contact time under the tongue and dry mouth [27]. Given the lack of dose proportionality, the exposure and the clinical response predicted in the current simulations represent the best-case scenario for the SL formulation. At the highest dose of apomorphine, the real exposure and the real clinical response is expected to be inferior to the reported values by a factor related to the less-proportional increase in the exposure with the increase of the apomorphine dose.

The results of this analysis also included greater mean AUEC, and shorter mean time to maximal response observed for the SC formulation compared to SL, while the incremental mean maximum response values for ascending doses of the two formulations were roughly comparable. The AUC was estimated over the interval of 0–90 min (Table 4), which corresponds to the duration of effect seen in clinical trials but may affect the interpretation of the results. Apomorphine administration can be repeated if needed (within 2 h intervals based on US prescribing information) [9, 10].

These results are generally consistent with a published head-to-head PK trial in individuals with Parkinson’s disease and OFF episodes that also showed higher C_max_ and shorter T_max_ with the SC compared to SL formulation [27]. That study did not report efficacy outcomes, and estimated exposure over 24 h (AUC_0-24 h_), whereas our analysis estimated exposure over the effectiveness window (AUC_0-90 min_) which may be more relevant for PK/PD comparisons. Still, a visual inspection of their data shows higher apomorphine exposure for the SC relative to SL formulation over the first 90-min post-dosing, which is consistent with our analysis [27].

Results of the current model also align with published clinical trials. Administration of apomorphine by SC injection may have advantages of providing a more reliable ON response (95% of OFF episodes aborted), whereas reported reliability of ON response has been lower and more variable with SL administration (35%–79%) [13, 22, 28, 29]. Similarly, the reported onset appeared faster with the SC formulation in clinical trials of SC (5–10 min) relative to trials of SL (15–30 min), with numerically larger reductions in UPDRS scores at early timepoints (SL: < 10 points at 15 min, SC: ~ 18 points at 15 min) [8, 13, 22, 29].

It has been suggested that differences in product PK profiles might translate into safety advantages for the SL formulation due to slower rise in plasma concentrations and lower peak concentrations [27]; data from a recently presented cross-over trial showed generally similar rates of common adverse events for the two formulations during initial titration and maintenance [30]. Conversely, clinical trial data suggest formulation-specific adverse events might be less well tolerated during longer-term use with the SL formulation. In a three-month study, oropharyngeal adverse events occurred in 31% of patients and led to discontinuation in 50% of individuals who experienced them [13]. For SC apomorphine, bruising (15%) or ecchymoses (10%) at the injection site were the most common injection-site adverse events in long-term apomorphine SC trials; these typically do not lead to discontinuation [9, 31]. With respect to ease of administration and preferences, hesitancy to inject is often brought up as a theoretical concern and patients in general prefer oral dosage forms; however, patients are more willing to self-inject than physicians may perceive if it poses potential for greater efficacy [32]. Apomorphine injection showed good patient acceptance in a long-term open-label trial with more than 50% of the 546 enrolled subjects remaining in treatment at 12 months, and > 75% administering apomorphine injections daily [31].

The principal limitation of our study was that it is based on modelled data, though as outlined above, our results show good alignment with clinical studies, and model-based PK/PD simulations are a common way to evaluate formulation dose–effect relationships for comparative analysis in the absence of head-to-head studies [24]. Additionally, we did not initially consider duration of response in modelling our analysis as both apomorphine SC and SL are indicated for the acute treatment of OFF episodes, and the primary goal of therapy is to provide immediate symptom relief until the patient’s regularly scheduled medication resumes effect. Based on a minimum clinically important change (MCIC) of just over 3-point improvement in UPDRS [23], the current model shows an approximate duration of effect for approved doses of SC apomorphine of 60 to 90 min, and just under 90 min for the approved 30-mg dose of SL apomorphine (Fig. 3). The 30-mg dose was the only approved SL dose to reach an MCIC in the current model. These estimates align with reported reliability and duration of effect in clinical studies.

The findings from this analysis can have important clinical implications, considering that the daily duration of off-time for patients with Parkinson’s disease can negatively impact their quality of life and that even a small change in the UPDRS motor scores can be clinically relevant [12, 23]. Specifically, time-to-on, or a latency from the treatment intake to the patient turning to on-state has been recognized as a major contributor to total daily off-time [11], and SC apomorphine treatment has been shown to rapidly and significantly reduce time-to-on in patients with Parkinson’s disease experiencing delayed onset of their morning levodopa dose [29], which is consistent with the present data. Therefore, the effectiveness of “on demand” therapy for motor fluctuations may play a critical role in patient’s wellbeing and potentially influence patient’s preference and clinician’s choice of treatment, as well as medication adherence.

## Conclusions

Faster onset of action was observed for the SC formulation compared to SL in reducing motor fluctuations measured with the UPDRS motor score. These data may be useful for clinicians when selecting an “on demand” treatment for patients with Parkinson’s disease experiencing motor fluctuations.

### Supplementary Information

Below is the link to the electronic supplementary material.

Supplementary file1 (DOCX 45 KB)

## Data Availability

The simulations in our analysis are based on previously published models; therefore, data are not available. Queries about the data should be directed to the corresponding author.

## References

[1] Chou KL, Stacy M, Simuni T, Miyasaki J, Oertel WH, Sethi K, Fernandez HH, Stocchi F (2018). The spectrum of "off" in Parkinson's disease: What have we learned over 40 years?. Parkinsonism Relat Disord.

[2] Ahlskog JE, Muenter MD (2001). Frequency of levodopa-related dyskinesias and motor fluctuations as estimated from the cumulative literature. Mov Disord.

[3] Hauser RA, Kremens DE, Elmer LW, Kreitzman DL, Walsh RR, Johnson R, Howard R, Nguyen JT, Patni R (2019). Prevalence of Dyskinesia and OFF by 30-Minute Intervals Through the Day and Assessment of Daily Episodes of Dyskinesia and OFF: Novel Analyses of Diary Data from Gocovri Pivotal Trials. J Parkinsons Dis.

[4] Pietz K, Hagell P, Odin P (1998). Subcutaneous apomorphine in late stage Parkinson's disease: a long term follow up. J Neurol Neurosurg Psychiatry.

[5] Hechtner MC, Vogt T, Zollner Y, Schroder S, Sauer JB, Binder H, Singer S, Mikolajczyk R (2014). Quality of life in Parkinson's disease patients with motor fluctuations and dyskinesias in five European countries. Parkinsonism Relat Disord.

[6] Stocchi F, Antonini A, Barone P, Tinazzi M, Zappia M, Onofrj M, Ruggieri S, Morgante L, Bonuccelli U, Lopiano L, Pramstaller P, Albanese A, Attar M, Posocco V, Colombo D, Abbruzzese G, group Ds (2014). Early DEtection of wEaring off in Parkinson disease: the DEEP study. Parkinsonism Relat Disord.

[7] Jenner P, Katzenschlager R (2016). Apomorphine - pharmacological properties and clinical trials in Parkinson's disease. Parkinsonism Relat Disord.

[8] Carbone F, Djamshidian A, Seppi K, Poewe W (2019). Apomorphine for Parkinson's Disease: Efficacy and Safety of Current and New Formulations. CNS Drugs.

[9] APOKYN® (2022). Prescribing information.

[10] KYNMOBI^TM^ (2020) Prescribing information. Shire US Inc, Marlborough, MA

[11] Merims D, Djaldetti R, Melamed E (2003). Waiting for ON: a major problem in patients with Parkinson disease and ON/OFF motor fluctuations. Clin Neuropharmacol.

[12] Perez-Lloret S, Negre-Pages L, Damier P, Delval A, Derkinderen P, Destee A, Meissner WG, Tison F, Rascol O, of the CSG (2017). L-DOPA-induced dyskinesias, motor fluctuations and health-related quality of life: the COPARK survey. Eur J Neurol.

[13] Olanow CW, Factor SA, Espay AJ, Hauser RA, Shill HA, Isaacson S, Pahwa R, Leinonen M, Bhargava P, Sciarappa K, Navia B, Blum D, investigators CTHS (2020). Apomorphine sublingual film for off episodes in Parkinson's disease: a randomised, double-blind, placebo-controlled phase 3 study. Lancet Neurol.

[14] Nicolle E, Pollak P, Serre-Debeauvais F, Richard P, Gervason CL, Broussolle E, Gavend M (1993). Pharmacokinetics of apomorphine in parkinsonian patients. Fundam Clin Pharmacol.

[15] Agbo F, Crass RL, Chiu YY, Chapel S, Galluppi G, Blum D, Navia B (2021). Population pharmacokinetic analysis of apomorphine sublingual film or subcutaneous apomorphine in healthy subjects and patients with Parkinson's disease. Clin Transl Sci.

[16] Gancher ST, Nutt JG, Woodward WR (1991). Absorption of apomorphine by various routes in parkinsonism. Mov Disord.

[17] Derendorf H, Meibohm B (1999). Modeling of pharmacokinetic/pharmacodynamic (PK/PD) relationships: concepts and perspectives. Pharm Res.

[18] U. S. Food and Drug Administration, Center for Drug Evaluation and Research. Approval Package for Application 21–264, Clinical Pharmacology and Biopharmaceutics Review Part 2. https://www.accessdata.fda.gov/drugsatfda_docs/nda/2004/21-264_Apokyn_BioPharmr_P2.pdf.

[19] U. S. Food and Drug Administration, Center for Drug Evaluation and Research. Approval Package for Application Number 21–264, Clinical Pharmacology and Biopharmaceutics Review Part 1, Submission Date October 17, 2003. https://www.accessdata.fda.gov/drugsatfda_docs/nda/2004/21-264_Apokyn_BioPharmr_P1.pdf.

[20] Fahn SER, UPDRS program members (1987). Recent developments in Parkinson's disease.

[21] Agbo F, Chiu YY, Chapel S, Navia B (2020). Exposure-response efficacy model of apomorphine sublingual film for the on-demand treatment of "OFF" episodes in patients with Parkinson's disease [Abstract]. Parkinsonism Relat Disord.

[22] Hauser RA, Olanow CW, Dzyngel B, Bilbault T, Shill H, Isaacson S, Dubow J, Agro A (2016). Sublingual apomorphine (APL-130277) for the acute conversion of OFF to ON in Parkinson's disease. Mov Disord.

[23] Horvath K, Aschermann Z, Acs P, Deli G, Janszky J, Komoly S, Balazs E, Takacs K, Karadi K, Kovacs N (2015). Minimal clinically important difference on the Motor Examination part of MDS-UPDRS. Parkinsonism Relat Disord.

[24] Holford NHG, Sheiner LB (1981). Understanding the Dose-Effect Relationship-Clinical Application of Pharmacokinetic-Pharmacodynamic Models. Clin Pharmacokin.

[25] Borkar N, Mu H, Holm R (2018). Challenges and trends in apomorphine drug delivery systems for the treatment of Parkinson's disease. Asian J Pharm Sci.

[26] Bilbault T, Taylor S, Walker R, Grundy SL, Pappert EJ, Agro A (2016). Buccal mucosal irritation studies of sublingual apomorphine film (APL-130277) in Syrian golden hamsters. Ther Deliv.

[27] Agbo F, Isaacson SH, Gil R, Chiu YY, Brantley SJ, Bhargava P, Navia B (2021). Pharmacokinetics and comparative bioavailability of apomorphine sublingual film and subcutaneous apomorphine formulations in patients with Parkinson's disease and "OFF" episodes: results of a randomized, three-way crossover, open-label study. Neurol Ther.

[28] Dewey RB, Hutton JT, LeWitt PA, Factor SA (2001). A randomized, double-blind, placebo-controlled trial of subcutaneously injected apomorphine for parkinsonian off-state events. Arch Neurol.

[29] Isaacson S, Lew M, Ondo W, Hubble J, Clinch T, Pagan F (2017). Apomorphine Subcutaneous Injection for the Management of Morning Akinesia in Parkinson's Disease. Mov Disord Clin Pract.

[30] Rascol O, Poewe W, Stocchi F, Chaudhuri R, Kassube J, Lopez Manzanares L, Leta V, Zhang I, Bowling A, Wu S, Pappert E (2022) Safety and tolerability of apomorphine sublingual film and subcutaneous apomorphine for the treatment of OFF episodes in Parkinson's disease. Movement Disorders 37:S348. 10.1002/mds.29223

[31] LeWitt PA, Ondo WG, Van Lunen B, Bottini PB (2009). Open-label study assessment of safety and adverse effects of subcutaneous apomorphine injections in treating "off" episodes in advanced Parkinson disease. Clin Neuropharmacol.

[32] Imamovic A, Melyan Z, Kasibhatla C, Kumar R (2021). “Needle Phobia” in Patients with Parkinson’s Disease (PD) Experiencing OFF Episodes is Uncommon (2356). Neurology.

