# Correction: Model-based comparison of subcutaneous versus sublingual apomorphine administration in the treatment of motor fluctuations in Parkinson’s disease

**DOI:** 10.1007/s10928-024-09923-w

**Published:** 2024-05-30

**Authors:** Azmi Nasser, Roberto Gomeni, Gianpiera Ceresoli-Borroni, Lanyi Xie, Gregory D. Busse, Zare Melyan, Jonathan Rubin

**Affiliations:** 1 Formerly with Supernus Pharmaceuticals, Inc., 9715 Key West Ave, Rockville, MD 20850 USA; 2 PharmacoMetrica, Lieu-dit Longcol, La Fouillade, France; 3 Supernus Pharmaceuticals, Inc., Rockville, MD USA

**Correction to: Journal of Pharmacokinetics and Pharmacodynamics**

10.1007/s10928-024-09914-x

In this article, the additional correction in Reference list and text citation of references in the article starting Ref 3 to Ref 29 provided by the author was inadvertently missed during the proof correction.

Also the author‘s correction to delete references 26 to 31 from the original version and addition of new reference was not incorporated in the reference list.

The references added retrospectively are given below for reference:

Hauser RA, Kremens DE, Elmer LW, Kreitzman DL, Walsh RR, Johnson R, Howard R, Nguyen JT, Patni R (2019) Prevalence of Dyskinesia and OFF by 30-Minute Intervals Through the Day and Assessment of Daily Episodes of Dyskinesia and OFF: Novel Analyses of Diary Data from Gocovri Pivotal Trials. J Parkinsons Dis 9 (3):591–600. doi:10.3233/JPD-181565.

Pietz K, Hagell P, Odin P (1998) Subcutaneous apomorphine in late stage Parkinson’s disease: a long term follow up. J Neurol Neurosurg Psychiatry 65 (5):709–716. doi:10.1136/jnnp.65.5.709.

Jenner P, Katzenschlager R (2016) Apomorphine - pharmacological properties and clinical trials in Parkinson’s disease. Parkinsonism Relat Disord 33 Suppl 1:S13-S21. doi:10.1016/j.parkreldis.2016.12.003.

Agbo F, Chiu YY, Chapel S, Navia B (2020) Exposure-response efficacy model of apomorphine sublingual film for the on-demand treatment of “OFF” episodes in patients with Parkinson’s disease [Abstract]. Parkinsonism Relat Disord 79:e85-86.

Agbo F, Isaacson SH, Gil R, Chiu YY, Brantley SJ, Bhargava P, Navia B (2021) Pharmacokinetics and Comparative Bioavailability of Apomorphine Sublingual Film and Subcutaneous Apomorphine Formulations in Patients with Parkinson’s Disease and “OFF” Episodes: Results of a Randomized, Three-Way Crossover, Open-Label Study. Neurol Ther 10 (2):693–709. doi:10.1007/s40120-021-00251-6.

Dewey RB, Jr., Hutton JT, LeWitt PA, Factor SA (2001) A randomized, double-blind, placebo-controlled trial of subcutaneously injected apomorphine for parkinsonian off-state events. Arch Neurol 58 (9):1385–1392. doi:10.1001/archneur.58.9.1385.

The original article has been corrected.

