# Supplementary material for: Model-based comparison of subcutaneous versus sublingual apomorphine administration in the treatment of motor fluctuations in Parkinson’s disease
Source: J Pharmacokinet Pharmacodyn. 2024 Apr 5;51(4):385–93. doi: 10.1007/s10928-024-09914-x (PMC11254985; doi:10.1007/s10928-024-09914-x)
Supplement: Supplementary file 1 — Supplementary file1 (DOCX 45 KB) [file 10928_2024_9914_MOESM1_ESM.docx]

**Journal of Pharmacokinetics and Pharmacodynamics**

**Model-Based Comparison of Subcutaneous versus Sublingual Apomorphine Administration in the Treatment of Motor Fluctuations in Parkinson’s Disease**

Azmi Nasser, PhD;^a^ Roberto Gomeni, PhD; Gianpiera Ceresoli-Borroni, PhD; Lanyi Xie, PhD; Gregory D. Busse, PhD; Zare Melyan, PhD; Jonathan Rubin, MD

^a^ Supernus Pharmaceuticals, Inc., Rockville, MD, USA

**Corresponding author:**

Azmi Nasser, PhD

Supernus Pharmaceuticals, Inc.

9715 Key West Ave

Rockville, MD 20850, USA

Email: [anasser@supernus.com](mailto:anasser@supernus.com)

Phone: 240-403-5303

ORCID ID: 0000-0003-0514-8410

**Supplemental Table 1** Descriptive statistics of the parameters qualifying the clinical response to each apomorphine dose at different levels of inter-individual variability

|  |  | **SC** | | | | **SL** | | | |
| --- | --- | --- | --- | --- | --- | --- | --- | --- | --- |
|  | **CV**, % | **Dose**, mg | **Parameter**,  mean ± SD | **95% CI** | **Median** | **Dose**, mg | **Parameter**,  mean ± SD | **95% CI** | **Median** |
| **Time to response**, min | 15 | 1 | 17.73 ± 3.14 | 16.21 – 19.24 | 17.8 | 20 | 44.82 ± 7.2 | 43.63 – 46.01 | 44.84 |
|  |  | 2 | 12.52 ± 3.20 | 12.24 – 12.81 | 11.94 | 30 | 38.52 ± 6.69 | 37.91 – 39.14 | 37.76 |
|  |  | 3 | 8.14 ± 1.57 | 8 – 8.27 | 7.88 | 40 | 33.38 ± 5.15 | 32.93 – 33.84 | 33.27 |
|  |  | 4 | 6.61 ± 1.22 | 6.51 – 6.72 | 6.44 | 50 | 30.19 ± 5.12 | 29.74 – 30.64 | 30.23 |
|  | 30 | 1 | 14.93 ± 5.06 | 13.78 – 16.08 | 14.61 | 20 | 39.88 ± 11.82 | 38.14 – 41.62 | 38.09 |
|  |  | 2 | 12.27 ± 5.33 | 11.76 – 12.79 | 10.98 | 30 | 36.82 ± 11.11 | 35.69 – 37.94 | 36.12 |
|  |  | 3 | 8.65 ± 3.60 | 8.33 – 8.97 | 7.8 | 40 | 33.4 ± 10.78 | 32.41 – 34.39 | 32.22 |
|  |  | 4 | 7.34 ± 3.34 | 7.04 – 7.63 | 6.57 | 50 | 30.53 ± 9.63 | 29.67 – 31.39 | 30.02 |
|  | 45 | 1 | 12.86 ± 5.62 | 11.80 – 13.92 | 11.96 | 20 | 37.84 ± 16.33 | 35.51 – 40.17 | 35.08 |
|  |  | 2 | 11.32 ± 6.13 | 10.67 – 11.96 | 10.05 | 30 | 33.97 ± 14.71 | 32.34 – 35.6 | 31.29 |
|  |  | 3 | 8.89 ± 5.06 | 8.43 – 9.35 | 7.53 | 40 | 32.72 ± 14.46 | 31.31 – 34.13 | 31.46 |
|  |  | 4 | 8.11 ± 5.32 | 7.63 – 8.59 | 6.48 | 50 | 31.04 ± 13.68 | 29.76 – 32.32 | 30.07 |
| **Duration of response**, min | 15 | 1 | 15.89 ± 8.78 | 11.65 – 20.12 | 13.98 | 20 | 34.05 ± 13.4 | 31.83 – 36.26 | 35.71 |
|  |  | 2 | 41.74 ± 11.74 | 40.7 – 42.79 | 41.88 | 30 | 49.17 ± 9.74 | 48.27 – 50.06 | 51.42 |
|  |  | 3 | 67.75 ± 10.12 | 66.86 – 68.64 | 67.63 | 40 | 56.42 ± 5.48 | 55.94 – 56.9 | 56.63 |
|  |  | 4 | 78.2 ± 7.23 | 77.57 – 78.84 | 81.8 | 50 | 59.81 ± 5.12 | 59.36 – 60.26 | 59.77 |
|  | 30 | 1 | 28.82 ± 16.49 | 25.07 – 32.56 | 25.91 | 20 | 43.76 ± 15.42 | 41.49 – 46.02 | 46.46 |
|  |  | 2 | 44.49 ± 19.18 | 42.64 – 46.35 | 43.17 | 30 | 48.98 ± 14.85 | 47.48 – 50.49 | 52.19 |
|  |  | 3 | 64.59 ± 17.05 | 63.08 – 66.11 | 66.98 | 40 | 55.4 ± 12.4 | 54.26 – 56.55 | 56.81 |
|  |  | 4 | 72.23 ± 14.69 | 70.94 – 73.52 | 79.31 | 50 | 58.63 ± 10.78 | 57.66 – 59.59 | 59.71 |
|  | 45 | 1 | 36.78 ± 20.94 | 32.84 – 40.72 | 32.73 | 20 | 46.7 ± 18.33 | 44.08 – 49.31 | 49.48 |
|  |  | 2 | 48.85 ± 21.97 | 46.53 – 51.17 | 47.72 | 30 | 52.03 ± 17.07 | 50.14 – 53.92 | 53.9 |
|  |  | 3 | 62.65 ± 20.31 | 60.8 – 64.51 | 66.56 | 40 | 55.5 ± 15.62 | 53.98 – 57.02 | 57.31 |
|  |  | 4 | 68.22 ± 19.05 | 66.51 – 69.92 | 77.05 | 50 | 57.28 ± 15.19 | 55.86 – 58.7 | 59.08 |
| **AUC_0-90_** | 15 | 1 | 66.36 ± 22.26 | 55.63 – 77.09 | 64.71 | 20 | 6.28 ± 2.04 | 5.94 – 6.62 | 5.62 |
|  |  | 2 | 129.2 ± 63.24 | 123.54 – 134.78 | 117.2 | 30 | 10.48 ± 4.58 | 10.06 – 10.9 | 9.64 |
|  |  | 3 | 297.4 ± 98.85 | 288.74 – 306.11 | 288.52 | 40 | 17.9 ± 6.84 | 17.3 – 18.5 | 17.16 |
|  |  | 4 | 436.8 ± 114.09 | 426.77 – 446.82 | 443.48 | 50 | 25.12 ± 8.2 | 24.4 – 25.84 | 24.42 |
|  | 30 | 1 | 109.16 ± 88.64 | 89.05 – 129.28 | 77.64 | 20 | 11.77 ± 11.86 | 10.03 – 13.52 | 8.27 |
|  |  | 2 | 176.98 ± 141.75 | 163.25 – 190.71 | 135.91 | 30 | 16.22 ± 17.18 | 14.48 – 17.96 | 12.02 |
|  |  | 3 | 321.12 ± 188.65 | 304.39 – 338.85 | 288.63 | 40 | 23.98 ± 23.23 | 21.84 – 26.12 | 18.44 |
|  |  | 4 | 428.95 ± 211.84 | 410.32 – 447.58 | 433.2 | 50 | 31.37 ± 29.4 | 28.75 – 34 | 24.29 |
|  | 45 | 1 | 165.80 ± 159.53 | 135.80 – 195.81 | 110.03 | 20 | 24.47 ± 48.07 | 17.61 – 31.34 | 11.96 |
|  |  | 2 | 239.97 ± 210.62 | 217.76 – 262.18 | 169.18 | 30 | 29.9 ± 51.24 | 24.23 – 35.57 | 16.15 |
|  |  | 3 | 355.51 ± 249.20 | 332.75 – 378.27 | 297.76 | 40 | 39.52 ± 64.23 | 33.27 – 45.77 | 21.02 |
|  |  | 4 | 438.67 ± 269.50 | 414.50 – 462.84 | 429.59 | 50 | 48.87 ± 73.35 | 42.01 – 55.72 | 26.65 |
| **Maximal response** | 15 | 1 | −4.03 ± 0.98 | −4.50 – −3.56 | −3.63 | 20 | −4.55 ± 1.22 | −4.75 – −4.35 | −4.16 |
|  |  | 2 | −7.76 ± 2.66 | −8 – −7.53 | −7.41 | 30 | −7.31 ± 2.67 | −7.56 – −7.07 | −6.88 |
|  |  | 3 | −14.68 ± 2.78 | −14.93 – −14.44 | −14.71 | 40 | −11.66 ± 3.33 | −11.95 – −11.37 | −11.51 |
|  |  | 4 | −18.56 ± 2.3 | −18.76 – −18.36 | −18.96 | 50 | −15.18 ± 3.17 | −15.46 – −14.9 | −15.39 |
|  | 30 | 1 | −6.05 ± 3.13 | −6.76 – −5.34 | −4.82 | 20 | −7.07 ± 3.47 | −7.58 – −6.56 | −5.94 |
|  |  | 2 | −9.12 ± 4.64 | −9.57 – −8.67 | −8.22 | 30 | −9.28 ± 4.85 | −9.77 – −8.79 | −8.17 |
|  |  | 3 | −14.46 ± 4.97 | −14.90 – −14.02 | −14.64 | 40 | −12.5 ± 5.31 | −12.99 – −12.01 | −11.84 |
|  |  | 4 | −17.48 ± 4.71 | −17.89 – −17.06 | −18.68 | 50 | −14.83 ± 5.46 | −15.31 – −14.34 | −15.35 |
|  | 45 | 1 | −7.99 ± 4.83 | −8.90 – −7.08 | −6.11 | 20 | −9.35 ± 5.25 | −10.1 – −8.6 | −7.76 |
|  |  | 2 | −10.77 ± 5.78 | −11.38 – −10.16 | −9.39 | 30 | −11.45 ± 6.03 | −12.11 – −10.78 | −10.19 |
|  |  | 3 | −14.64 ± 6.05 | −15.19 – −14.08 | −14.93 | 40 | −13.54 ± 6.23 | −14.14 – −12.93 | −13.09 |
|  |  | 4 | −16.92 ± 5.94 | −17.45 – −16.39 | −18.62 | 50 | −15.19 ± 6.42 | −15.79 – −14.59 | −15.98 |
| **Time to maximal response**, min | 15 | 1 | 25.58 ± 2.71 | 24.27 – 26.89 | 24 | 20 | 59.58 ± 5.94 | 58.6 – 60.56 | 60 |
|  |  | 2 | 26.49 ± 3.06 | 26.22 – 26.76 | 24 | 30 | 60.3 ± 5.88 | 59.76 – 60.84 | 60 |
|  |  | 3 | 26.45 ± 3.16 | 26.17 – 26.73 | 24 | 40 | 60.68 ± 5.74 | 60.18 – 61.19 | 60 |
|  |  | 4 | 26.52 ± 3.11 | 26.25 – 26.79 | 24 | 50 | 60.59 ± 5.74 | 60.08 – 61.09 | 60 |
|  | 30 | 1 | 25.06 ± 5.35 | 23.85 – 26.27 | 24 | 20 | 59.93 ± 12.33 | 58.12 – 61.75 | 60 |
|  |  | 2 | 26.49 ± 5.68 | 25.94 – 27.04 | 24 | 30 | 60.02 ± 11.36 | 58.87 – 61.17 | 60 |
|  |  | 3 | 26.91 ± 5.65 | 26.41 – 27.42 | 24 | 40 | 61.77 ± 11.62 | 60.7 – 62.84 | 60 |
|  |  | 4 | 27.09 ± 5.75 | 26.59 – 27.6 | 24 | 50 | 61.39 ± 11.7 | 60.34 – 62.44 | 60 |
|  | 45 | 1 | 25.14 ± 7.48 | 23.73 – 26.55 | 24 | 20 | 60.22 ± 16.5 | 57.87 – 62.57 | 60 |
|  |  | 2 | 26.36 ± 8 | 25.52 – 27.21 | 24 | 30 | 59.43 ± 15.44 | 57.72 – 61.14 | 60 |
|  |  | 3 | 27.16 ± 8.42 | 26.39 – 27.93 | 24 | 40 | 62.43 ± 15.59 | 60.91 – 63.94 | 60 |
|  |  | 4 | 27.76 ± 9.02 | 26.95 – 28.57 | 24 | 50 | 62.32 ± 15.93 | 60.83 – 63.81 | 60 |

Abbreviations: AUC_0-90_, area under the response curve for 0 to 90 minutes; CI, confidence interval; CV, coefficient of variation; min, minute; SC, subcutaneous; SD, standard deviation; SL, sublingual
